# Cost Analysis of Screening Programmes for Developmental Dysplasia of the Hip: A Systematic Review

**DOI:** 10.1007/s43465-021-00501-7

**Published:** 2021-09-06

**Authors:** Philip Harper, Rohit Gangadharan, Daryl Poku, Alexander Aarvold

**Affiliations:** 1 Department of Paediatric Orthopaedic Surgery, Southampton Children’s Hospital, Southampton, UK; 2 Department of Trauma and Orthopaedics, University Hospitals Dorset, Bournemouth, UK; 3 University of Southampton, Southampton, UK

**Keywords:** Developmental dysplasia of the hip, Screening, Cost, Ultrasound, Clinical examination

## Abstract

**Aims:**

The aim of this study was to assess screening costs in developmental dysplasia of the hip (DDH), to provide any clarity on the cost-effectiveness of various hip screening programmes internationally.

**Methods:**

A PROSPERO-registered systematic review was performed by examining cost analysis studies of various DDH screening programmes, including those based around clinical examination, selective ultrasound and universal ultrasound. Costs were analysed using narrative synthesis.

**Results:**

There were 14 studies included in this review. Two studies found that clinical hip screening is advantageous over no screening at all, both in terms of overall cost and favourable outcomes. When considering selective ultrasound imaging *versus* clinical screening, two studies found it to be more expensive, one found it cheaper and three studies calculated the overall programme costs to be similar. With universal ultrasound, four studies calculated this to be cheaper than clinical or selective ultrasound screening due to a reduced late detection and surgery rate. However, a comparable number of studies concluded that the increased financial costs of universal ultrasound were greater than the reduction in surgical costs. No studies included any long-term data.

**Conclusion:**

There is a dearth of information on DDH screening costs, with significant heterogeneity amongst the existing literature. Future research should include the cost analysis of long-term complications of DDH, including the social and psychological impact of early onset arthritis, as well as gender specific ultrasound screening programmes.

## Introduction

Developmental dysplasia of the hip (DDH) is a spectrum of disease ranging from mild acetabular dysplasia with a stable hip to complete dislocation with abnormal acetabular and femoral morphology. It affects up to 1% of live births and represents a significant public health issue globally [1, 2]. The benefits of early diagnosis and treatment are evident, with conservative measures such as Pavlik Harness splinting usually resulting in complete resolution of symptoms and normal growth and development thereafter [3]. If, on the other hand, DDH goes undetected in the first few months of life then surgery may be required—mainly closed or open reduction with or without osteotomy of femur or pelvis. Surgical treatment can be associated with less favourable outcomes, higher complication rates and longer hospital stays. As such, newborn hip screening has been a topic of debate for a number of decades regarding the optimum strategy for early DDH detection.

Various screening programmes exist around the world, which include the following: clinical examination only (Ortolani and Barlow manoeuvres), selective ultrasound for those with abnormal clinical examination findings or risk factors, and finally universal ultrasound of all newborns [4]. There is scepticism regarding the effectiveness of screening programmes which rely on clinical examination alone as signs may be too subtle or perhaps even absent in early life [5]. On the contrary, ultrasound may lead to the over-diagnosis and subsequent over treatment of DDH as many cases of immature hips will self-resolve without intervention [6, 7]. The economic impact of such screening programmes is multifactorial and requires careful consideration by governments and policymakers. Costs incurred with initial screening may be offset by savings associated with earlier diagnosis, favourable outcomes and avoidance of surgery. The physical and psychological benefits to newborns who receive early diagnoses and treatment are prominent and must also be considered, but are difficult to objectively measure in a research setting.

This systematic review was conducted to provide as much clarity as currently exists on the cost-effectiveness of DDH screening programmes, as calculated in a number of previous observational studies. This would hope to inform both policy-makers on DDH screening and research avenues.

## Methods

### Search Strategy and Study Selection

**Fig. 1:**
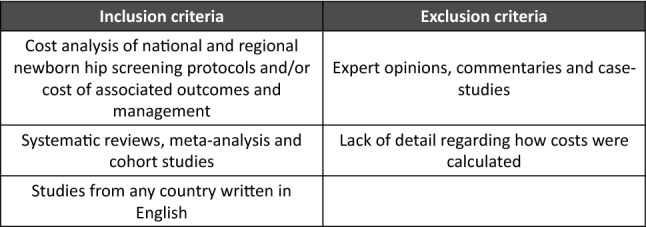
Inclusion and exclusion criteria

The review was registered with PROSPERO (the international prospective register of systematic reviews) prior to its commencement. A literature search was conducted using PubMed, MEDLINE, Web of Science, EMBASE, CINAHL and reference lists of chosen articles. Within the databases, keywords including ‘hip’, ‘screening’, ultrasound’ and ‘cost’ were used to narrow the search. Only studies written in English were included and no unpublished studies were sought. An initial search was carried out prior to starting the review with a re-run prior to final analysis. Studies were selected by two authors independently and screened by an additional author with any disagreements resolved consensually. Full-text papers were reviewed in every case and references were downloaded into the Endnote reference management software. Inclusion and exclusion criteria are outlined in Fig. 1.

### Quality Assessment

Two authors independently conducted quality assessment to highlight any key strengths or flaws of selected studies, along with potential bias.

### Data Extraction

The following data were extracted: country of study, year of publication, study time-period, number/gender/age of infants, calculated costs of screening programme including initial radiology, staffing, hospital stay and outpatient costs along with subsequent follow-up and complications.

### Outcome Measures

Various forms of newborn hip screening exist in most developed countries; however, there is a lack of consensus regarding the most cost-effective screening method. Investment in thorough screening at an early age may offset later costs by minimising complications through late or missed diagnoses. Therefore, the primary outcome was to evaluate direct and indirect costs associated with various hip screening programmes.

### Statistical Analysis

Evaluations of economic data and results were combined using narrative synthesis.

## Results

**Fig. 2:**
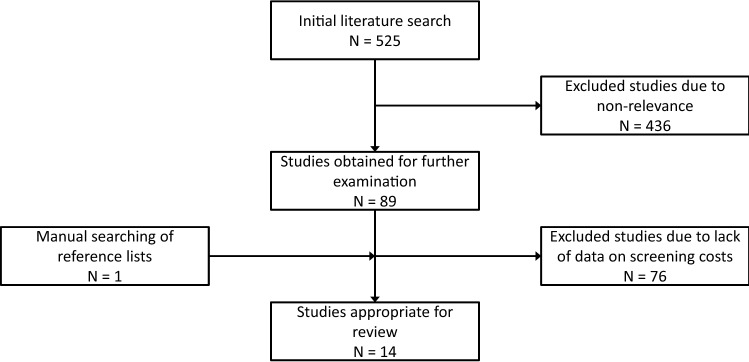
Flowchart demonstrating the study selection process

The initial literature search generated 525 studies from the aforementioned databases. Of these, 89 were deemed at least partially relevant and their abstracts and full-texts were further examined. One additional study was added after searching the reference lists [8]—initially missed as there was no indication of screening costs in its title. After applying the eligibility criteria, 14 full text studies remained and were included in this review (Fig. 2). The key components included in each of these 14 studies are summarised in Table 1. Studies were excluded primarily due to little or no mention of DDH screening costs (Fig. 1).

**Table 1:** Elements of cost analysis in each study

Study	Clinical screening	Selective ultrasound screening	Universal ultrasound screening	Inpatient hospital costs	Outpatient hospital costs	Conservative management	Surgical management	Long-term management and follow up of complications
Fulton et al. [9] Vancouver, Ca	Yes	No	No	Yes	Yes	Yes	Yes	No
Tredwell [10] Vancouver, Ca	Yes	No	No	Yes	Yes	Yes	Yes	No
Paton et al. [8] Blackburn, UK	No	No	No	No	No	No	No	No
Preiss et al. [12] Dundee, Scotland	Yes	Yes	No	Yes	No	Yes	No	No
Kumar et al. [13] India	No	Yes	No	No	No	No	No	No
Elbourne et al. [14] UK and Ireland	Yes	No	Yes	Yes	Yes	Yes	Yes	No
Gray et al. [15] UK	Yes	No	Yes	Yes	Yes	Yes	Yes	No
Clegg et al. [16] Coventry, UK	Yes	Yes	Yes	Yes	No	Yes	Yes	No
Woodacre et al. [17] Devon, UK	No	Yes	Yes	Yes	Yes	Yes	Yes	No
Brown et al. [18] UK	Yes	Yes	Yes	Yes	Yes	Yes	Yes	No
Thaler et al. [20] Austria	Yes	No	Yes	Yes	Yes	Yes	Yes	No
Bralic et al. [21] Croatia	No	No	Yes	Yes	Yes	Yes	Yes	No
Rosendahl et al. [22] Norway	Yes	Yes	Yes	Yes	Yes	Yes	Yes	No
Geitung et al. [23] Norway	No	No	Yes	Yes	Yes	Yes	Yes	No

## Clinical Screening Versus No Screening

There appears to be no dispute in the literature regarding the cost-effectiveness of clinical screening versus no hip screening programme at all. In Vancouver, Fulton et al. [9] calculated that the cost of not screening was Can $13,700/1000 infants, based on surgery being required on expected late diagnosed cases. The cost of clinical screening was Can $7,225 / 1000 infants which also included the likely cost of missed diagnoses despite screening. This figure was based on an estimated incidence of 14.5/1000 infants of total DDH cases.

Also in British Columbia, Tredwell [10] found considerable economic benefit of a clinical screening programme versus no screening assuming that the false negative rate of clinical examination falls below 1.23/1000 births. The authors calculated a cost benefit of Can $15,717/1000 births with clinical screening on the basis of 100% detection rate and zero false-negative rate. This is likely an overestimation of the cost saving as the rate of missed diagnosis on clinical examination is widely acknowledged [11].

## Selective Ultrasound Versus Clinical Screening

Paton et al. [8] calculated a rate of 0.87/1000 births for infants requiring surgery in a selective ultrasound screening programme, similar to robust clinical screening. Whilst the authors concluded that a national selective ultrasound screening programme cannot be justified based on cost or clinical outcomes, no specific cost analysis was outlined to justify this statement. Preiss et al. [12] argue that the cost of screening cannot be judged on rate of open surgery alone. In Scotland, two methods of hip screening were compared between 1994 and 2000, namely clinical screening *versus* selective ultrasound. This resulted in a small decrease in rates of open surgery from 0.51/1000 to 0.42/1000 respectively and a clinically significant 50% reduction in the requirement of closed reductions and spica immobilisation. It is postulated that targeted ultrasound screening may be more cost-effective when all aspects of cost are included, thereby contradicting the findings by Paton et al.

In India, Kumar et al. [13] studied a cohort of infants between 2006 and 2014 who all received a clinical hip screen between 36 and 48 h after birth. Infants received an ultrasound scan before discharge if they had an abnormal clinical examination finding, or at 6 and 12 weeks if they had a DDH risk factor. There were 736 babies in total who were scanned, 20 of which had abnormal sonographic findings. Of these 20 babies, 18 had immature hips which were managed conservatively using double nappies to maintain abduction and all had resolved by the 12-week scan. The remaining two infants (which equates to a DDH incidence of 2.7/1000) had Types IIC and IIIB dysplasia based on Graf’s classification and were managed with Pavlik Harness instead of double nappies. Both failed Pavlik Harness treatment and thus required surgery; one had closed reduction at 6 months and the other had open reduction with femoral osteotomy at 16 months. As each scan cost 200 Indian Rupees (INR), the cost of ultrasound scanning 736 infants amounted to INR 147,200. The authors observed that this was a similar cost to two hip replacements, which would otherwise be likely in young adulthood had these two infants not received DDH-correcting surgery. Similarly, had the 18 infants with immature hips not been detected early, there may have been a requirement for more intensive and costly treatment in later life. Currently in India, there is no national hip screening programme. This paper highlights that universal clinical screening with selective ultrasound is perhaps justified and is unlikely to be more expensive than the long-term cost of late/undiagnosed DDH cases.

## Universal Ultrasound Versus Other Screening Methods

Most of the existing literature on cost analysis of screening methods relates to comparisons against actual or projected universal ultrasound programmes. A number of UK-based studies exist in the literature, plus analyses from Austria, Norway, Croatia and India.

Elbourne et al. [14] performed a multicentre randomised trial across 33 centres in the UK and Ireland (UK Hip Trial) to evaluate the cost of a universal ultrasound screening programme. There were 629 infants randomised to a universal ultrasonography group and a clinical examination group (of which ultrasound was used in confirmed cases after initial splinting). The cost of ultrasound in the ultrasonography group was £42/patient compared to £23 in the clinically diagnosed group. Total costs, however, were £102 less per patient in the ultrasonography group due to savings made from lower rates of surgery, fewer radiographs, outpatient visits and days spent in hospital compared to the clinical examination group. Gray et al. [15] built on these findings and found that the mean difference in cost was US$190 more expensive per patient in the clinical examination group of which the main contributing factor was an increase in the number of days spent in hospital. The same conclusion was, therefore, drawn that the increase in cost of ultrasound per patient was offset by an even bigger reduction in other costs.

Clegg et al. [16] analysed and compared the cost of surgical treatment with three different DDH screening programmes carried out at different time periods in Coventry: clinical examination alone (A, 1976–1986), selective ultrasound screening of those with abnormal clinical examination findings and/or risk factors (B, 1986–1989) and universal ultrasound screening of all newborns (C, 1989–1996). Group A had the highest number of operations and group C the least. This equated to surgical costs of £5110/1000 births in group A, £3811/1000 births in group B and £468/1000 births in group C taking into account length of hospital admission, cost of the operating theatre and surgical team, implants, radiology and blood products. The total annual cost of surgery, Pavlik harness treatment and screening were then combined—Group A £22,188, group B £21,837 and group C £26,564 demonstrating the significant cost of ultrasound. The cost of ultrasound screening per child at that time was £6 per patient.

Woodacre et al. [17] compared the cost of the regional selective ultrasound programme in Devon, England, against alternative screening strategies between 1997 and 2008: ultrasound scanning all newborns, and ultrasound scanning all girls and at risk boys. Alternative programmes were modelled based on the cost of each individual screening component. The cost of selective ultrasound screening across this 11-year period was found to be £104,000 per annum. Of this annual cost, 73% (or £76,000) was spent on the 99.5% of infants with normal hips. The remaining 27% (or £28,000) was spent on the remaining 0.5% of infants with dysplastic hips. The cost of treating those with late-diagnosed DDH was seven times higher than the cost of early treatment with a Pavlik Harness. However, in those that failed Pavlik Harness treatment and required further management, the costs incurred were twelve times higher. The annual cost of alternative screening programmes was projected to be £162,000 when ultrasound scanning all girls and at-risk boys, and £280,900 with scanning all newborns. This increase in cost is attributed to the increase in ultrasound scanning, despite the decrease in surgical intervention. However, the requirement for increased radiological equipment, personnel and training for such programmes was not included in the cost analysis. The cost of ultrasound screening each child was £12—double the cost at the time of the calculations by Clegg et al. in the previous decades.

Prior to the introduction of selective ultrasound screening in the UK, Brown et al. [18] estimated that universal ultrasound screening would cost £31,000,000 per annum based on 700,000 births per year versus £21,000,000 for selective ultrasound screening and £7,000,000 for clinical screening. It is suggested, however, that clinical examination screening becomes as effective as, and less costly than, selective ultrasound screening when carried out by experienced examiners. However, the additional cost of these dedicated practitioners was not accounted for in this cost analysis.

Austria introduced a nationwide universal ultrasound screening programme between 1983 and 1988. Prior to this, screening was reliant on clinical examination and ultrasound was only used when the diagnosis was unclear [19]. Thaler et al. [20] compared the cost of investigation and management of DDH in two cohorts either side of the introduction of their universal ultrasound-based screening programme (period 1: 1978–1982, period 2: 1993–1997). There was a significant decrease in the number of interventions in the universal ultrasound programme, for example, the rate of splinting dropped from an average of 170 cases per year to 90 per year. Similarly, there was a decrease in the rate of surgery from a mean of 17.8–2.6 per year. It can be deduced that clinical examination alone was leading to significant over-treatment. The overall cost of screening and treatment combined was €57,000/year higher in the ultrasound group primarily due to the costs associated with universal ultrasound. When analysing the cost of treatment alone, there was a significant cost reduction after the introduction of the universal ultrasound programme from €410,000 to €117,000 owing to fewer operative and non-operative treatments.

A Croatian study [21] estimated that if all children born in 1996 had received ultrasound screening at 1 month of age, the total cost including treatment would have been US$338,241. This included the cost of ultrasound, along with the training of all existing neonatologists in its use. Out of a total of 1046 DDH cases that year, 622 were diagnosed late (> 3 months) after clinical signs became more apparent. The cost of all those babies starting treatment after 3 months was calculated at US$533,578. Late treatment costs were therefore 1.6 times higher when ultrasound was not utilised. This increase in cost was explained by longer and more complex treatment and rehabilitation costs when DDH was detected late. The authors suggest that each neonatologist would need 71.4 h per year to implement a universal ultrasound screening programme. However, it begs the question whether these physicians could do this without leaving gaps in other aspects of their work, thereby creating a necessity for additional clinicians—a potential cost which had not been considered.

In Norway, Rosendahl et al. [22] calculated the cost of screening to be $16 per infant with universal ultrasound, $7 with selective ultrasound and $6 with clinical screening. However, the total cost of screening plus treatment was $27.90 per infant with universal ultrasound screening compared to $29.60 and $29.20 for selective ultrasound and clinical screening, respectively. Universal ultrasound resulted in a higher cost of harness treatment; however, the total cost was calculated to be lower than the other screening protocols due to earlier diagnoses and zero cases requiring surgery for late detection.

Geitung et al. [23] projected a cost analysis of a national universal ultrasound programme based on treatment costs for 26 patients with late diagnosed DDH (1984–85) in two Norwegian Hospitals. The cost of universal ultrasound in this region of Norway was calculated at NOK (Norwegian Kroner) 1,375,000. This included the training, equipment, administrative costs and expected higher harness treatment rates, minus the cost-saving due to zero expected late-diagnosed cases. When scaled to a national level, this equated to NOK 13,750,000 plus an additional NOK 4.5 million on physician training. The authors suggest that the overall net cost of universal ultrasound cannot be justified with Norway’s publicly funded healthcare system based on the small number of late detected cases that would be prevented.

## Discussion

Considering the high prevalence of DDH, the significant healthcare costs of screening and treatment and the variety of screening programmes that exist, it is remarkable how little there is in the literature on cost benefit analyses. The most robust analyses to date are now summarised in this systematic review. Unfortunately, there is variability in the methods used and what elements are costed. Perhaps this explains why there are conflicting conclusions drawn across these studies.

Furthermore, the studies in this review have been conducted at various times over a forty-year period, with different currencies and in different healthcare settings. Given the variability of exchange rates, it is almost impossible to compare costs between different studies and to apply said costs to today’s economic climate. As such, it was more appropriate to compare relative screening programme costs within each study (using one currency at any one point in time). The research by Kumar et al. [13] is the only published paper on hip screening costs to come out of a developing country. There is a need for further detailed cost-analyses in countries with different healthcare and economic models. Policymakers will need to calculate cost analysis based on locally prevalent factors such as national or regional DDH incidence, availability of resources and trained personnel, ultrasound cost and cost of conservative and surgical treatment. In doing this, screening programmes can be tailored to a specific region or country.

Based on existing literature, clinical hip screening appears to be advantageous over no screening at all, both in terms of overall cost and favourable outcomes [9, 10]. Conflicting views become apparent when considering if, and how, ultrasound should be used. Two studies indicate that selective ultrasound cannot be justified over clinical screening based on cost or clinical outcomes [8, 18], one found a cost benefit of a selective ultrasound programme [12] and three studies calculated the overall programme costs to be similar [13, 16, 22]. The persistently high late detection rate in the UK despite the introduction of selective ultrasound screening in 1986 would further question the economic value of this programme [11].

With a dramatically reduced late detection (and surgery) rate in a system with universal ultrasound, some studies have calculated this to be cheaper than clinical or selective ultrasound screening [14, 15, 21, 22]. However, a comparable number of studies have found the increased costs of universal ultrasound outweigh the reduction in surgical costs [16–18, 20, 23]. The lack of consensus in the literature is thus clearly demonstrated.

Interestingly, the study by Woodacre et al. [17] provides a calculation of the cost of ultrasound scanning all girls and at risk boys. Given the heavily weighted prevalence of DDH in the female population, this may be a more attractive screening programme in the eyes of policymakers as it would be less of an upfront cost than universal ultrasound. This option has been suggested previously [22].

Various research groups have expressed concerns that universal ultrasound screening would result in over-treatment [3, 24], however, Clegg et al. [16] report that the intervention rate with universal screening was 0.4%, which was comparable to the intervention rate with selective screening shown in a study by Vedantam et al. in Sheffield 1995 [25]. Two Austrian studies [19, 20] report that clinical examination screening is the main culprit when it comes to overtreatment. Overtreatment exposes infants to certain risks associated with hip abduction splinting [26, 27]. This systematic review has unearthed yet more ambiguity within the management of DDH, by highlighting the doubt as to which programme would actually be most at risk of over treatment. Austria have continued a universal ultrasound screening programme despite Thaler et al. [20] calculating its increased cost—due to the significant reduction in conservative treatment, hospital admission and surgeries rather than looking at cost alone.

An important gap in the literature exists in that zero studies included the cost of long-term complications of DDH in their cost analysis, such as early onset osteoarthritis and a requirement for joint replacement at a young age [28, 29]. Ashraf et al. [30] found that the average cost of total hip replacement was US$450 higher per patient in a cohort of adults with a history of hip dysplasia compared to a group with primary osteoarthritis—a limitation being that that some of the arthritis cohort may have had concomitant hip dysplasia. The increase in cost in the DDH group was attributed to higher operative and implant costs owing to more complex procedures. As these operations are more likely to be performed at a younger age, the cost of revision surgeries and follow up are also likely to be significant. Furthermore, the social costs of time off work, reduced income, reduced taxation and societal benefits are not factored into any study, but would be more in any programme whereby the late detection rate is higher. Moreover, litigation is increasing [31] but has yet to be considered in any cost analysis of DDH screening and treatment. If these factors were included in a cost analysis of screening programmes, universal ultrasound may appear more favourable.

## Conclusion

A small number of cost-analysis studies on DDH screening programmes have been conducted over the past 40 years. There is significant heterogeneity between these studies, not least as a result of different healthcare systems, economies, patient populations, availability of resources and trained personnel. There is no long-term data and there is no easy way of quantifying the psychological and social benefits to a person in whom the long-term sequelae of DDH have been prevented by screening and early detection. Whilst cost analysis is a critical tool in informing policy-making, there is a dearth of this data in DDH. Future research should include the cost analysis of long-term complications of DDH, as well as gender specific ultrasound screening programmes.
